# Associations of chronic diarrheal symptoms and inflammatory bowel disease with sleep quality: A secondary analysis of NHANES 2005–2010

**DOI:** 10.3389/fneur.2022.858439

**Published:** 2022-08-24

**Authors:** Jingyun Zhang, Senhai Yu, Gang Zhao, Xiaoyan Jiang, Yimin Zhu, Zuyun Liu

**Affiliations:** ^1^Center for Clinical Big Data and Analytics, Second Affiliated Hospital and Department of Big Data in Health Science School of Public Health, The Key Laboratory of Intelligent Preventive Medicine of Zhejiang Province, Zhejiang University School of Medicine, Hangzhou, China; ^2^Jinhua Town Community Health Service Center, Hangzhou, China; ^3^Center for Disease Control and Prevention, Hangzhou, China; ^4^Key Laboratory of Arrhythmias, Ministry of Education, Department of Pathology and Pathophysiology, School of Medicine, Tongji University, Shanghai, China; ^5^Department of Epidemiology and Biostatistics, School of Public Health, Zhejiang University School of Medicine, Hangzhou, China

**Keywords:** chronic diarrheal symptoms, inflammatory bowel disease, sleep quality, sleep disorder, sleep trouble

## Abstract

**Objective:**

Poor sleep quality is highly prevalent in patients with chronic diarrheal symptoms or inflammatory bowel disease (IBD). This study aimed to evaluate the associations of chronic diarrheal symptoms and IBD with sleep quality in the general US population.

**Methods:**

14,696 adults (≥20 years) from the National Health and Nutrition Examination Survey (2005–2010) were included in the study. Chronic diarrheal symptoms and IBD were defined by self-reports. Sleep quality was assessed by sleep disorder, sleep trouble, and sleep duration. Multivariable logistic regression models were used to examine the associations.

**Results:**

After adjustment of a series of covariates, we found that participants with chronic diarrheal symptoms or IBD had higher odds of sleep disorder [chronic diarrheal symptoms: odds ratio (OR) = 1.20, 95% confidence interval (CI) = 1.04–1.38; IBD: OR = 3.86, 95% CI = 1.92–7.77] and sleep trouble (chronic diarrheal symptoms: OR = 1.19, 95% CI = 1.09–1.30; IBD: OR = 2.32, 95% CI = 1.30–4.14), respectively. Sleep duration for participants with IBD was significantly shorter than that for those without IBD (β = −0.39, 95% CI = −0.78 to 0.01, *P* = 0.045). Subgroup analyses revealed that the associations of chronic diarrheal symptoms and IBD with sleep disorder and sleep trouble were more pronounced among women.

**Conclusions:**

In this large sample of US adults, we found that chronic diarrheal symptoms and IBD were significantly associated with sleep quality, particularly in women. The findings highlight the importance of managing bowel health to promote high quality of sleep; and thus, improve quality of life in this subpopulation.

## Introduction

Chronic diarrhea is common in all age groups, affecting 6.6% of the population in the US (1). Chronic diarrhea is the most prevalent symptom in irritable bowel syndrome (IBS) and inflammatory bowel disease (IBD). IBS is a complex, functional gastrointestinal disorder characterized by chronic abdominal pain or discomfort and altered bowel habits (2). The majority of IBS patients present chronic diarrhea, resulting in a major subtype of IBS, terms as IBS-D (diarrhea predominant IBS). IBD is an idiopathic inflammatory disorder of gastrointestinal tract, including Crohn's Disease (CD) and ulcerative colitis (UC) (3). Recently, IBD has become a global disease with increased prevalence worldwide. Chronic diarrhea and the two diseases (i.e., IBS and IBD) cause fatigue and depression, affect quality of life, and increase the risk of hospitalizations and death (4–6).

Poor sleep quality has been increasingly recognized as one of the most important factors that lead to the low quality of life. Sleep quality has been measured by one or more terms in literature, including circadian rhythm, insomnia, hypersomnia, parasomnia, narcolepsy, nightmare, restless leg syndrome, sleep initiation, sleep maintenance, sleep duration (7, 8). Poor sleep quality causes deteriorations in both physical and psychological health such as fatigue, anxiety, and stress (9). Thus, it has been suggested that sleep quality is also a crucial indicator of life quality. One of the factors associated with poor sleep quality is chronic disease (10, 11). In fact, studies have reported that in comparison with healthy controls, persons with IBD have poorer overall sleep quality, and significantly greater sleep trouble and disruption (12, 13). Poor sleep quality is highly prevalent in IBD patients, in both UC and CD (14–19). Previous studies have also showed that the prevalence of poor sleep quality was higher in IBS patients relative to healthy controls (20, 21) and different locations (or area), age, gender, and occupations may affect the sleep quality in IBS patients (22). Surprisingly, few studies have specifically examined the relationship between sleep quality and chronic diarrhea, the most and common symptom in IBS and IBD. With the increasing numbers of adults affected by chronic diarrhea, findings from such studies would be informative to the better management of this symptom. Furthermore, previous studies on either IBS or IBD are limited due to the relatively small sample size and the issue on sample representativeness. For example, to date, no studies have utilized a nationally representative dataset to evaluate the association of sleep quality with IBD, making the generalizability of the findings unclear.

In this study, using data from the National Health and Nutrition Examination Survey (NHANES), we aimed to evaluate the associations of chronic diarrheal symptoms and IBD with sleep quality (measured by sleep disorder, sleep trouble, and sleep duration) in the general US population aged 20 years and older. Given the influence of age and gender on sleep quality, we examined whether the association of chronic diarrheal symptoms and IBD with sleep quality differed by age and gender. The findings could be of importance to the management of bowel health to maximize the quality of life in the US population.

## Methods

### Study population

The sample used was from three cross-sectional surveys of NHANES (2005–2006, 2007–2008, and 2009–2010). NHANES is an ongoing program with the aim of assessing the health and nutritional status of US children and adults by the National Center for Health Statistics. The sample in the NHANES is selected to represent the general population of all ages in counties across the US. All participants visit the physician, and dietary interviews and body measurements are included for everyone. More details are provided on the official website of NHANES (https://www.cdc.gov/nchs/nhanes/index.htm). All participants provided informed consent. And NHANES is approved by the National Center for Health Statistics Research Ethics Review Board (Protocol #2005-06, Continuation of Protocol #2005-06).

In this study, we excluded participants with missing data on sleep quality (*N* = 64) and bowel health (*N* = 2,372), leaving a final analytic sample of 14,696 participants aged 20 years and older. As the question on IBD was only available in the 2009–2010 survey, the analysis on IBD was limited to 4,356 out of 14,696 participants who had data on IBD (almost all participants from 2005 to 2010 had data on sleep quality).

### Measures

#### Chronic diarrheal symptoms and IBD

As described in a previous study (4), a participant was defined as having chronic diarrheal symptoms if she/he reported having symptoms of bowel leakage (gas, mucus, and liquid) two or more times a week. That being said, we used bowel leakage symptoms as an alternative indicator of chronic diarrhea as done in previous studies (4). A participant was defined as having IBD if she/he reported a diagnosis of UC or CD.

#### Sleep quality

Sleep quality was assessed using three dimensional questions, as done in previous studies (23). Participants were asked if she/he has been ever told by a doctor or other health professional that she/he has trouble sleeping. Those participants whose response was “yes” were defined as having sleep trouble. Similarly, when participants were asked whether a doctor or other health professional had told him or her that she/he has sleep disorder, those who answered “yes” were defined as having sleep disorder. Participants were also asked that “how much sleep do you get (hours)?” and the sleep duration was then documented.

#### Covariates

We considered covariates as following: age, race/ethnicity (non-Hispanic white, non-Hispanic black, Hispanic, and others), education levels, family socioeconomic status, marital status (currently married/living with partner, and others), body mass index (BMI), smoking status, and alcohol consumption. Education levels, smoking status and alcohol consumption were classified accordance to a previous study (24). For example, education levels were defined as less than high school (HS), HS/general educational development (GED), having attended college but not receiving at least a bachelor's degree (some college), and having a bachelor's degree or higher (college). Smoking status was divided into never smokers, former smokers, and current smokers. And alcohol consumption was merged into none in past year, <1 drink per month, 1–3 drinks per month, and ≥1 drink per week. Family socioeconomic status was stratified into three categories on the basis of family poverty-income ratio: low income (poverty-income ratio, <1.3), middle income (≥1.3 and <3.5), and high income (≥3.5) (25). BMI was calculated as weight in kilograms divided by height in meters squared. The criteria of underweight, normal, overweight and obese were BMI < 18.5 kg/m^2^, 18.5 ≤ BMI < 25.0 kg/m^2^, 25.0 ≤ BMI < 30.0 kg/m^2^, and BMI ≥ 30.0 kg/m^2^, respectively. We also considered chronic diseases because of their potential influence on sleep quality, including diabetes, hypertension, congestive heart failure, myocardial infarction, stroke, chronic bronchitis, emphysema, and cancer.

### Statistical analyses

We presented the basic characteristics of the study population using mean ± standard deviation (SD) for continuous variables or No. (percentages) for categorical variables in total and by chronic diarrheal symptoms and IBD, respectively.

To investigate the associations of chronic diarrheal symptoms and IBD with sleep quality, we first presented the No. and proportions of sleep disorder and sleep trouble, as well as the mean ± SD of sleep duration by chronic diarrheal symptoms and IBD, respectively. The Chi-square test and *t*-test were used for the comparisons. For sleep disorder and sleep trouble, we then used multivariable logistic regression models and documented odds ratios (ORs) and corresponding 95% confidence intervals (CIs) from two models. Model 1 adjusted for age and gender. Model 2 further adjusted for race/ethnicity, current marital status, education, body mass index, family socioeconomic status, smoking status, and drinking status. For sleep duration, we used linear regression models and documented coefficients and 95% CIs from the two similar models as above.

To examine whether the association of chronic diarrheal symptoms and IBD with sleep quality differed by age and gender, we conducted subgroup analyses according to age and gender. However, the results of IBD may not be robust because the number of IBD was extremely small (*n* = 56 in 4,356 participants).

To assess the robustness of the results, we repeated the main analysis by additionally adjusting for chronic diseases including hypertension, diabetes, congestive heart failure, myocardial infarction, stroke, emphysema, chronic bronchitis, and cancer. We considered two-sided *P*-value < 0.05 to be statistically significant. Analyses were performed using SAS 9.4 (SAS Institute, Cary, NC).

## Results

### Basic characteristics of the study participants

Table 1 shows the basic characteristics of the study population. A total of 4,053 (27.6%) and 56 (1.3%) participants had chronic diarrheal symptoms and IBD, respectively. The mean age of the 14,696 adults was 49.4 years, and about half of the sample were women (50.9%). Young and middle-aged adults (<60 y) accounted for 66.6%. Participants with chronic diarrheal symptoms were more likely to be older, women, non-Hispanic White, currently married, obese, and have chronic diseases. Participants with IBD were more likely to be older, former smoker, overweight, and have some chronic diseases (e.g., hypertension, stroke and chronic bronchitis).

**Table 1:** Basic characteristics of the study population.

**Characteristic**	**Overall**	**Chronic diarrheal symptoms^a^**			**Inflammatory bowel disease^b^**		
	**(*N* = 14,696)**	**With**	**Without**	***P*-value**	**With**	**Without**	***P*-value**
		**(*N* = 4,053)**	**(*N* = 10,643)**		**(*N* = 56)**	**(*N* = 4,300)**	
**Age, years**	49.4 ± 18.0	50.8 ± 17.9	48.9 ± 18.1	0.601	51.8 ± 13.1	44.0 ± 14.2	0.480
<60 years	9,789 (66.6)	2,586 (63.8)	7,203 (67.7)	**<0.001**	35 (62.5)	3,473 (80.8)	**<0.001**
≥60 years	4,907 (33.4)	1,467 (36.2)	3,440 (32.3)		21 (37.5)	827 (19.2)	
**Gender**				**0.016**			0.120
Men	7,213 (49.1)	1,924 (47.5)	5,289 (49.7)		22 (39.3)	2,139 (49.7)	
Women	7,483 (50.9)	2,129 (52.5)	5,354 (50.3)		34 (60.7)	2,161 (50.3)	
**Race/ethnicity**				**<0.001**			0.374
Non-hispanic white	7,233 (49.2)	2,199 (54.3)	5,034 (47.3)		26 (46.4)	1,937 (45.1)	
Non-hispanic black	2,918 (19.9)	682 (16.8)	2,236 (21.0)		6 (10.7)	793 (18.4)	
Mexican American	2,713 (18.5)	712 (17.6)	2,001 (18.8)		15 (26.8)	858 (20.0)	
Others	1,832 (12.5)	460 (11.4)	1,372 (12.9)		9 (16.1)	712 (16.6)	
**Marital status**				**0.016**			0.510
Currently married/living with partner	9,001 (61.3)	2,546 (62.9)	6,455 (60.7)		32 (57.1)	2,640 (61.5)	
Others	5,687 (38.7)	1,505 (37.2)	4,182 (39.3)		24 (42.9)	1,656 (38.6)	
**Education^c^**				0.250			0.055
HS	4,152 (28.3)	1,124 (27.8)	3,028 (28.5)		18 (32.1)	1,135 (26.4)	
HS/GED	3,521 (24.0)	989 (24.4)	2,532 (23.8)		5 (8.9)	999 (23.3)	
Some college	4,067 (27.7)	1,092 (27.0)	2,975 (28.0)		22 (39.3)	1,252 (29.2)	
College	2,942 (20.0)	846 (20.9)	2,096 (19.7)		11 (19.6)	907 (21.1)	
**Socioeconomic status**				0.556			0.378
Low income (PIR <1.3)	4,026 (29.6)	1,094 (29.2)	2,932 (29.7)		13 (25.0)	1,336 (34.0)	
Middle income (1.3 ≤ PIR <3.5)	5,253 (38.6)	1,433 (38.2)	3,820 (38.7)		20 (38.5)	1,390 (35.4)	
High income (PIR ≥ 3.5)	4,346 (31.9)	1,222 (32.6)	3,124 (31.6)		19 (36.5)	1,204 (30.6)	
**Smoking status**				0.645			**0.044**
Never smokers	7,718 (52.5)	2,128 (52.5)	5,590 (52.5)		24 (42.9)	2,352 (54.7)	
Former smokers	3,737 (25.4)	1,048 (25.9)	2,689 (25.3)		19 (33.9)	883 (20.5)	
Current smokers	3,236 (22.0)	875 (21.6)	2,361 (22.2)		13 (23.2)	1,065 (24.77)	
**Drinking status**				0.397			0.822
None in past year	2,966 (20.2)	849 (21.0)	2,117 (19.9)		9 (16.1)	684 (15.9)	
<1 drink per month	11,268 (76.8)	3,069 (75.8)	8,199 (77.1)		46 (82.1)	3,445 (80.1)	
1–3 drinks per month	319 (2.2)	94 (2.3)	225 (2.1)		1 (1.8)	114 (2.7)	
≥1 drink per week	138 (0.9)	37 (0.9)	91 (0.9)		0 (0.0)	56 (1.3)	
**BMI** ^**d**^				**0.024**			**0.025**
Underweight	358 (2.4)	110 (2.7)	248 (2.3)		3 (5.4)	82 (1.9)	
Normal weight	3,959 (26.9)	1,046 (25.8)	2,913 (27.4)		9 (16.1)	1,130 (26.3)	
Overweight	4,998 (34.0)	1,347 (33.2)	3,651 (34.3)		26 (46.4)	1,410 (32.8)	
Obese	5,381 (36.6)	1,550 (38.2)	3,831 (36.0)		18 (32.1)	1,678 (39.0)	
**Chronic disease**			
Diabetes	1,698 (11.6)	545 (13.5)	1,153 (10.9)	**<0.001**	9 (16.1)	416 (9.7)	0.109
Hypertension	5,078 (34.6)	1,512 (37.4)	3,566 (33.6)	**<0.001**	33 (58.9)	1,213 (28.3)	**<0.001**
Congestive heart failure	462 (3.2)	144 (3.6)	318 (3.0)	**0.080**	0 (0.0)	68 (1.6)	0.343
Myocardial infarction	633 (4.3)	206 (5.1)	427 (4.0)	**0.004**	2 (3.6)	117 (2.7)	0.700
Stroke	545 (3.7)	181 (4.5)	364 (3.4)	**0.003**	5 (8.9)	98 (2.3)	**0.001**
Chronic bronchitis	867 (5.9)	284 (7.0)	583 (5.5)	**<0.001**	7 (12.5)	199 (4.6)	**0.006**
Emphysema	344 (2.3)	115 (2.8)	229 (2.2)	**0.014**	1 (1.8)	70 (1.6)	0.927
Cancer	1,400 (9.5)	459 (11.3)	941 (8.9)	**<0.001**	6 (10.7)	270 (6.3)	0.177

PIR, Poverty income ratio; BMI, body mass index; PIR, poverty-income ratio.

Values are given as mean ± standard deviation (SD) or No. (percentages). Percentages may not sum to 100 because of rounding. There were missing data on marital status (n = 8), education (n = 14), socioeconomic status (N = 1,071), smoking status (n = 5), drinking status (n = 15), diabetes (n = 13), hypertension (n = 24), congestive heart failure (n = 46), myocardial infarction (n = 25), stroke (n = 23), chronic bronchitis (n = 31), emphysema (n = 15), and cancer (n = 16).

a Having symptoms of chronic diarrhea consisting of gas, mucus, and liquid 2 or more times a week was considered to have symptoms.

b Inflammatory bowel disease was defined as either a diagnosis of ulcerative colitis or Crohn's Disease.

c Education levels included: HS, less than high school; HS/GED, HS/general educational development; some college, having attended college but not receiving at least a bachelor's degree; college, having a bachelor's degree or higher.

^d^BMI was calculated as weight in kilograms divided by height in meters squared. Underweight was defined as BMI < 18.5 kg/m^2^; normal was defined as 18.5 ≤ BMI < 25.0 kg/m^2^; overweight was defined as 25.0 ≤ BMI < 30.0 kg/m^2^; and obese was defined as BMI ≥ 30.0 kg/m^2^. P < 0.05 was marked by bold font.

### Chronic diarrheal symptoms, IBD and sleep quality

Table 2 shows that 8.9 and 27.0% of participants with chronic diarrheal symptoms reported that they had sleep disorder and trouble. The corresponding proportions were 7.0 and 22.5% in those without chronic diarrheal symptoms, with statistically significant differences (all *P* < 0.001). There was no significant difference in sleep duration between participants with and without chronic diarrheal symptoms (*P* = 0.334). IBD patients had poor sleep quality, indicated by the significant higher proportions of sleep disorder (23.2 vs. 7.2%) and sleep trouble (46.4 vs. 24.3%) and shorter sleep duration (6.3 vs 6.8 h), compared to those without IBD (All *P* < 0.05).

**Table 2:** Chronic diarrheal symptoms, inflammatory bowel disease, and sleep quality.

**Sleep quality**	**Chronic diarrheal symptoms**			**Inflammatory bowel disease**		
	**With**	**Without**	***P*-value**	**With**	**Without**	***P*-value**
	**(*N* = 4,053)**	**(*N* = 10,643)**		**(*N* = 56)**	**(*N* = 4,300)**	
**Sleep disorder**			<0.001			<0.001
Yes	361 (8.9)	750 (7.0)		13 (23.2)	309 (7.2)	
No	3,692 (91.1)	9,893 (93.0)		43 (76.8)	3,991 (92.8)	
**Sleep trouble**			<0.001			<0.001
Yes	1,095 (27.0)	2,394 (22.5)		26 (46.4)	1,046 (24.3)	
No	2,958 (73.0)	8,249 (77.5)		30 (53.6)	3,254 (75.7)	
**Sleep duration**	6.8 ± 1.5	6.8 ± 1.4	0.334	6.3 ± 1.6	6.8 ± 1.4	0.009

Values are given as mean ± standard deviation (SD) or No. (percentages).

The Chi-square test was applied for the comparisons of sleep disorder and sleep trouble, and the t-test was applied for the comparisons of sleep duration.

### Associations of chronic diarrheal symptoms and IBD with sleep quality

Table 3 presents the associations between chronic diarrheal symptoms and IBD with sleep quality, based on logistic regression models for sleep disorder and sleep trouble and linear regression models for sleep duration. Overall, persons with chronic diarrheal symptoms and IBD had higher odds of sleep disorder by 26% (OR = 1.26, 95% CI = 1.11–1.44) and 224% (OR = 3.24, 95% CI = 1.71–6.13), respectively, relative to their counterparts without that symptom (after adjusting for age and gender). For sleep trouble, the corresponding ORs were 1.24 (95% CI = 1.14–1.34) and 2.23 (95% CI = 1.30–3.82). When further adjusted for race/ethnicity, current marital status, education, body mass index, family socioeconomic status, smoking status, and drinking status, we found consistent results. For example, chronic diarrheal symptoms and IBD were associated with a 20% (OR = 1.20, 95% CI = 1.04–1.38) and 286% (OR = 3.86, 95% CI = 1.92–7.77) higher odds of sleep disorder, respectively. We found that the sleep duration for participants with IBD were significantly shorter than that for those without IBD (*P* = 0.015), while there was no significant difference between participants with and without chronic diarrheal symptoms (*P* = 0.487). Further adjustment did not change the results substantially (*P* = 0.045 and 0.920, respectively).

**Table 3:** Associations of chronic diarrheal symptoms and inflammatory bowel disease with sleep quality.

	**Sleep disorder**	**Sleep trouble**	**Sleep duration**
	**OR (95% CI)**	**OR (95% CI)**	**β (95% CI)**
**With chronic diarrheal symptoms**
Model 1	**1.26 (1.11, 1.44)**	**1.24 (1.14, 1.34)**	0.02 (−0.03, 0.07))
Model 2	**1.20 (1.04, 1.38)**	**1.19 (1.09, 1.30)**	0.00 (−0.05, 0.06)
**With inflammatory bowel disease**
Model 1	**3.24 (1.71, 6.13)**	**2.23 (1.30, 3.82)**	**−0.46 (−0.84**, **−0.09)**
Model 2	**3.86 (1.92, 7.77)**	**2.32 (1.30, 4.14)**	–**0.39 (**–**0.78**, –**0.01)**

OR, odds ratio; CI, confidence interval.

As described in Methods, we used logistic regression models for sleep disorder and sleep trouble and linear regression models for sleep hours. Model 1 adjusted for age and gender. Model 2 further adjusted for race/ethnicity, current marital status, education, body mass index, family socioeconomic status, smoking status, and drinking status. Effect sizes with P < 0.05 were marked by bold font.

In addition to the covariates in Model 2, we further adjusted for hypertension, diabetes, congestive heart failure, myocardial infarction, stroke, emphysema, chronic bronchitis, and cancer and provided the results in Supplementary Table S1. Overall, the results were maintained, such that chronic diarrheal symptoms and IBD were associated with 15% (OR = 1.15, 95% CI = 1.00–1.33) and 204% (OR = 3.04, 95% CI = 1.48–6.25) higher risks of sleep disorder, respectively. The association of IBD with sleep duration became nonsignificant but the direction remained the same.

### Associations of chronic diarrheal symptoms and IBD with sleep quality by age and gender

Results of the associations of chronic diarrheal symptoms and IBD with sleepquality in the subgroup analysis are shown in Table 4. We found that the associations were more pronounced in women. For example, women with chronic diarrheal symptoms had higher odds of sleep disorder by 30% (OR = 1.30, 95% CI = 1.07–1.59, Model 2) relative to their counterparts without this symptom. Meanwhile, we did not observe the significant association in men (OR = 1.09, 95% CI = 0.89–1.33). The associations of chronic diarrheal symptoms with sleep trouble differed little by either age or gender. Similar results were observed for IBD.

**Table 4:** Associations of chronic diarrheal symptoms and inflammatory bowel disease with sleep quality.

	**Chronic diarrheal symptoms**			**Inflammatory bowel disease**		
	**Sleep disorder**	**Sleep trouble**	**Sleep duration**	**Sleep disorder**	**Sleep trouble**	**Sleep duration**
	**OR (95% CI)**	**OR (95% CI)**	**β (95% CI)**	**OR (95% CI)**	**OR (95% CI)**	**β (95% CI)**
**Young and middle-aged adults (<60 years)**						
Model 1	**1.16 (0.98, 1.38)**	**1.21 (1.09, 1.35)**	**0.06 (0.00, 0.12)**	**3.86 (1.75, 8.50)**	**2.77 (1.39, 5.53)**	−0.17 (−0.65, 0.31)
Model 2	**1.13 (0.94, 1.35)**	**1.17 (1.04, 1.31)**	0.05 (−0.01, 0.11)	**4.76 (1.97, 11.51)**	**2.77 (1.30, 5.91)**	−0.21 (−0.69, 0.27)
**Older adults (≥60 years)**
Model 1	**1.40 (1.14, 1.73)**	**1.26 (1.10, 1.44)**	−0.03 (−0.13, 0.07)	2.41 (0.79, 7.36)	1.61 (0.66, 3.95)	−0.65 (−1.32, 0.02)
Model 2	**1.29 (1.03, 1.62)**	**1.22 (1.05, 1.41)**	−0.06 (−0.16, 0.04)	**4.23 (1.24, 14.38)**	1.95 (0.76, 4.99)	–**0.70 (**–**1.39**, –**0.01)**
**Men**
Model 1	**1.17 (0.98, 1.41)**	**1.24 (1.09, 1.41)**	0.06 (−0.02, 0.14)	1.61 (0.47, 5.60)	1.00 (0.36, 2.74)	−0.07 (−0.67, 0.53)
Model 2	**1.09 (0.89, 1.33)**	**1.20 (1.05, 1.37)**	0.04 (−0.04, 0.12)	3.04 (0.82, 11.12)	1.27 (0.45, 3.57)	–**0.11 (**–**0.11**, –**0.11)**
**Women**
Model 1	**1.36 (1.13, 1.64)**	**1.23 (1.10, 1.38)**	−0.01 (−0.09, 0.07)	**4.66 (2.17, 10.01)**	**3.50 (1.73, 7.08)**	–**0.53 (**–**1.04**, –**0.02)**
Model 2	**1.30 (1.07, 1.59)**	**1.19 (1.05, 1.33)**	−0.03 (−0.11, 0.05)	**4.77 (2.05, 11.13)**	**3.39 (1.58, 7.27)**	–**0.52 (**–**1.03**, –**0.01)**

OR, odds ratio; CI, confidence interval. As described in Methods, we used logistic regression models for sleep disorder and sleep trouble. Model 1 adjusted for age and gender. Model 2 further adjusted for race/ethnicity, current marital status, education, body mass index, family socioeconomic status, smoking status, and drinking status. Effect sizes with P < 0.05 were marked by bold font.

The P-values for interaction were 0.218 for the interaction term of age and sleep disorder, 0.667 for the interaction term of age and sleep disorder, and 0.030 for the interaction term of age and sleep duration among participants with chronic diarrheal symptoms based on Model 2.

The P-values for interaction were 0.496 for the interaction term of age and sleep disorder, 0.104 for the interaction term of age and sleep disorder, and 0.057 for the interaction term of age and sleep duration among participants with inflammatory bowel disease based on Model 2.

The P-values for interaction were 0.283 for the interaction term of gender and sleep disorder, 0.897 for the interaction term of gender and sleep disorder, and 0.205 for the interaction term of gender and sleep duration among participants with chronic diarrheal symptoms based on Model 2.

The P-values for interaction were 0.451 for the interaction term of gender and sleep disorder, 0.103 for the interaction term of gender and sleep disorder, and 0.030 for the interaction term of gender and sleep duration among participants with inflammatory bowel disease based on Model 2.

## Discussion

To the best of our knowledge, this study is the first one to focus on bowel health and sleep quality in a large nationally representative sample. The main finding of this study was that chronic diarrheal symptoms and IBD were significantly associated with sleep disorder and sleep trouble in US adults. Notably, the association of chronic diarrheal symptoms with sleep disorder was more pronounced in women.

The findings of the association of chronic diarrheal symptoms and IBD with sleep quality in this study are generally consistent with that from previous studies. For example, many studies have revealed that sleep disorder was significantly associated with IBS (26–29) and IBD (14, 30–34). Nevertheless, the association of total sleep duration with IBS has not reached a consensus. For instance, as observed in our study, the non-significant difference in sleep duration between persons with or without IBS was also reported in previous literature (35–37). There are a few exceptions. Based on data from nine IBD patients and seven healthy controls, Keefeer et al. (38) suggested a shorter sleep time among persons with IBD. This observation was also reported in another study with 24 IBD patients and 15 healthy controls (39). There might be at least two explanations for the conflicting results here. First, the significant association in the two studies above might be biased by the small sample size. Second, given the similar sleep patterns observed between IBS and IBD patients (14, 38, 40), it is highly possible that persons with chronic diarrheal symptoms have shorter sleep duration as do for IBD patients, which could be confounded by the other symptoms this group have. This confusion could be clarified when detailed evaluations of gastrointestinal symptoms are done in future studies. Finally, the physiological mechanisms underlying the observed association of chronic diarrheal symptoms and IBD with sleep quality are not quite clear, and might be related to inflammatory activity (high C-reactive protein) (15, 41).

We found that the more pronounced association of chronic diarrheal symptoms with sleep disorder in women. Our results added more evidence to findings from previous studies that have observed that the prevalence of sleep disorder in women with chronic diarrheal symptoms was higher than that in men (21, 42, 43). A potential explanation might be that sex hormones were different, and women had greater pain perception, which leads to differences in responses to sleep behaviors when getting chronic diarrheal (44). Another popular explanation might be the difference in gut microbiota in men and women with chronic diarrheal symptoms (45). Regarding the age difference, to the best of our knowledge, no previous studies have examined the association of sleep disorder with chronic diarrheal symptoms among older adults while comparing it to that in young adults. Our findings call for that much more attention should be paid to women, a highly vulnerable group in the society. The comparable results for the association of chronic diarrheal symptoms with sleep trouble by gender imply us that there might be some potential differences in sleep disorder and sleep trouble. This is beyond the scope of this study, but it would be of interest to explore in the future.

Our study has important implications. First, considering the large proportions of adults with bowel health issues, such as chronic diarrhea and IBD, and their relations to sleep quality, it is of importance for the public, medical staff, and family members to care for this subpopulation. This is particularly crucial taking the privacy of these symptoms into account. Second, for patients with sleep problems, medical staff may need to examine bowel health such as chronic diarrheal symptoms and IBD. Third, the findings call for more thoughts about the psychological mechanism between gastrointestinal disorder and sleep quality. For example, as mentioned before, chronic diarrhea always causes fatigue and depression. It remains unclear but interesting that whether depression serves as one possible way to link the two outcomes (chronic diarrhea and sleep disorder) together.

## Strengths and limitations

A major strength of this study is the large sample size of the study population, strengthening the generalizability of the findings. The large sample size also allowed us to perform subgroup analyses for chronic diarrheal symptoms (although the only 56 IBD cases impeded our subgroup analysis for IBD). In addition, we used three outcomes (sleep disorder, sleep trouble, and sleep duration) simultaneously to assess sleep quality, strengthening the findings and adding more evidence to previous studies. However, this study has several limitations to be acknowledged. First, the cross-sectional design of this study limited the causal inference between bowel health and sleep quality. Second, although a large sample size was utilized, we still had a small number of IBD patients, considering the relatively low prevalence of IBD, which suggested that the results for IBD should be interpreted carefully and further validation using larger sample size is needed. Third, the assessments of bowel health and sleep quality were based on self-reports, which might not be accurate but serves as the only conventional way in large epidemiological investigations. Moreover, more information on sleep pattern were not available. Fourth, we did not have more detailed information on chronic diarrhea (e.g., the duration), making it impossible to distinguish subtypes of the symptoms, which may have differentiated associations with sleep quality. In addition, there were no data on medication for sleep, mood disorder and gastrointestinal symptoms, which might be potential confounders.

## Conclusion

In summary, in this large sample of US adults, we found that chronic diarrheal symptoms and IBD were significantly associated with sleep quality and the associations were more pronounced in women. The findings highlight the importance of managing bowel health in daily life, particularly for women.

## Data availability statement

Publicly available datasets were analyzed in this study. This data can be found here: Centers for Disease Control and Prevention (CDC) National Health and Nutrition Examination Survey (NHANES), https://wwwn.cdc.gov/nchs/nhanes/Default.aspx, 2005–2006, 2007–2008, and 2009–2010.

## Author contributions

Conceptualization was proposed by XJ, YZ, and ZL. The methodological design and data analyses were conducted by JZ and ZL. Data collection and preparation were performed by SY and GZ. The results were interpreted by GZ, SY, and XJ. The first draft of the manuscript was written by JZ. XJ, YZ, and ZL provided overall supervision. All authors reviewed and edited the manuscript. All authors contributed to the article and approved the submitted version.

## Funding

This research was supported by fundings from Key Laboratory of Intelligent Preventive Medicine of Zhejiang Province (2020E10004) and Zhejiang University Global Partnership Fund (188170-11103). The funders had no role in the study design; data collection, analysis, or interpretation; in the writing of the report; or in the decision to submit the article for publication.

## Conflict of interest

The authors declare that the research was conducted in the absence of any commercial or financial relationships that could be construed as a potential conflict of interest.

## Publisher's note

All claims expressed in this article are solely those of the authors and do not necessarily represent those of their affiliated organizations, or those of the publisher, the editors and the reviewers. Any product that may be evaluated in this article, or claim that may be made by its manufacturer, is not guaranteed or endorsed by the publisher.
