# Supplementary material for: Associations of chronic diarrheal symptoms and inflammatory bowel disease with sleep quality: A secondary analysis of NHANES 2005–2010
Source: Front Neurol. 2022 Aug 24;13:858439. doi: 10.3389/fneur.2022.858439 (PMC9449577; doi:10.3389/fneur.2022.858439)
Supplement: Supplementary file 1 [file Table_1.docx]

**Table S1. Associations of chronic diarrheal symptoms and inflammatory bowel disease with sleep quality**

|  | **Sleep disorder** | **Sleep trouble** | **Sleep duration** |
| --- | --- | --- | --- |
|  | **OR (95% CI)** | **OR (95% CI)** | **β (95% CI)** |
| **With chronic diarrheal symptoms** | | | |
| Model 3+chronic disease | **1.15 (1.00, 1.33)** | **1.16 (1.06, 1.27)** | 0.01 (-0.05, 0.06) |
| **With inflammatory bowel disease** | | | |
| Model 3+chronic disease | **3.04 (1.48, 6.25)** | **1.83 (1.01, 3.30)** | -0.33 (-0.72, 0.05) |

Note: OR, odds ratio; CI, confidence interval; SE, standard error. As described in Methods, we used logistic regression models for sleep disorder and sleep trouble and generalized linear regression models for sleep hours. Here in addition to the covariates in Model 2, we further adjusted for hypertension, diabetes, congestive heart failure, myocardial infarction, stroke, emphysema, chronic bronchitis, and cancer. Effect sizes with P < 0.05 were marked by bold font.
